# The interaction between the perception of danger from coronavirus and the severity of burnout syndrome in medical workers during the COVID-19 pandemic in Russia

**DOI:** 10.1192/j.eurpsy.2021.823

**Published:** 2021-08-13

**Authors:** J. Koniukhovskaia, E. Pervichko, O. Mitina, O. Stepanova, E. Dorokhov

**Affiliations:** 1 Psychology, Lomonosov Moscow State University, Moscow, Russian Federation; 2 Faculty Of Psychology, Lomonosov Moscow State University, Moscow, Russian Federation; 3 Faculty Of Psychology And Social Sciences, Pirogov Russian National Research Medical University, Moscow, Russian Federation

**Keywords:** COVID-19, medical workers, burnout, perception of pandemic COVID-19

## Abstract

**Introduction:**

The COVID-19 pandemic has become a major challenge for both the overall health system and the individual ability for health professionals to stress coping.

**Objectives:**

To find the link between the perception of danger from coronavirus and the severity of burnout syndrome in medical workers during the COVID-19 pandemic in Russia.

**Methods:**

We used a socio-demographic questionnaire (20 questions), a Stress Perception Questionnaire (Linville, 1987; Ababkov et al., 2016), a Modified Pandemic Perception Questionnaire (Broadbent et al.,2006;Yaltonsky et al., 2017), and Maslach Burnout Inventory (Maslach et al., 1996; Lozinskaya et a. 2007). 249 medical workers (58 men and 191 women) took part in the online survey between April 27 and October 26 in Russia.

**Results:**

The severity of stress is positively correlated with the perception of pandemic as threatening (r=0.532, p=0.000) and unknown disease (r=0.297, p=0.000). Stress severity also correlates with all burnout parameters: exhaustion (r=0.737, p=0.000), depersonalization (r=0.342, p=0.000), and belief in personal achievement (r=-0.417, p=0.000). The perception of pandemics as threatening events is significantly associated with exhaustionm (r=0.458, p=0.000), depersonalization (r=0.133, p= 0.036), and belief in personal achievement (r=-0.152, p=0.016).The feeling of uncertainty from the pandemic is statistically significantly associated with exhaustion (r=0.242, p=0.000), while the feeling of control over the pandemic is positively associated with belief in personal achievements (r=0.129, p=0.042) and negatively associated with exhaustion (r=-0.161, p=0.011) and depersonalization (r=-0.125, p=0.049).

**Conclusions:**

Uncertainty and a sense of threat from the coronavirus and the pandemic are significant factors of stress and emotional burnout for health workers. To determine the interaction between parameters such as signs of burnout, perception of the COVID-19 pandemic and the severity of stress, further construction of a structural model is required.

